# Enhanced risk of record-breaking regional temperatures during the 2023–24 El Niño

**DOI:** 10.1038/s41598-024-52846-2

**Published:** 2024-02-29

**Authors:** Ning Jiang, Congwen Zhu, Zeng-Zhen Hu, Michael J. McPhaden, Deliang Chen, Boqi Liu, Shuangmei Ma, Yuhan Yan, Tianjun Zhou, Weihong Qian, Jingjia Luo, Xiuqun Yang, Fei Liu, Yuejian Zhu

**Affiliations:** 1 State Key Laboratory of Severe Weather (LASW), Chinese Academy of Meteorological Sciences, Beijing, 100081 China; 2 Climate Prediction Center, NCEP/NWS/NOAA, College Park, MD USA; 3 NOAA/Pacific Marine Environment Laboratory, Seattle, WA USA; 4 Department of Earth Sciences, University of Gothenburg, 40530 Gothenburg, Sweden; 5 State Key Laboratory of Numerical Modeling for Atmospheric Sciences and Geophysical Fluid Dynamics, Institute of Atmospheric Physics, Chinese Academy of Sciences, Beijing, 100029 China; 6 University of Chinese Academy of Sciences, Beijing, 100049 China; 7 Department of Atmospheric and Oceanic Sciences, Peking University, Beijing, 100871 China; 8 Guangzhou Institute of Tropical and Marine Meteorology/Guangdong Provincial Key Laboratory of Regional Numerical Weather Prediction, China Meteorological Administration, Guangzhou, 510641 China; 9 Institute for Climate and Application Research (ICAR), Nanjing University of Information Science and Technology, Nanjing, 210044 China; 10 School of Atmospheric Sciences, Nanjing University, Nanjing, 210023 China; 11 Key Laboratory of Tropical Atmosphere-Ocean System Ministry of Education, and Southern Marine Science and Engineering Guangdong Laboratory, School of Atmospheric Sciences Sun Yat-Sen University, Zhuhai, 519082 China; 12 Earth System Modeling and Prediction Centre (CEMC), China Meteorological Administration, Beijing, 100081 China

**Keywords:** Atmospheric science, Climate change

## Abstract

In 2023, the development of El Niño is poised to drive a global upsurge in surface air temperatures (SAT), potentially resulting in unprecedented warming worldwide. Nevertheless, the regional patterns of SAT anomalies remain diverse, obscuring where historical warming records may be surpassed in the forthcoming year. Our study underscores the significant influence of El Niño and the persistence of climate signals on the inter-annual variability of regional SAT, both in amplitude and spatial distribution. The likelihood of global mean SAT exceeding historical records, calculated from July 2023 to June 2024, is estimated at 90%, contingent upon annual-mean sea surface temperature anomalies in the eastern equatorial Pacific exceeding 0.6 °C. Regions particularly susceptible to recording record-high SAT include coastal and adjacent areas in Asia such as the Bay of Bengal and the South China Sea, as well as Alaska, the Caribbean Sea, and the Amazon. This impending warmth heightens the risk of year-round marine heatwaves and escalates the threat of wildfires and other negative consequences in Alaska and the Amazon basin, necessitating strategic mitigation measures to minimize potential worst-case impacts.

## Introduction

Changes in global surface air temperature (SAT) are influenced by external forcing (e.g., greenhouse gases) and internal climate variations^1,2^. The El Niño–Southern Oscillation (ENSO) is the strongest year-to-year determinant of climate variation on the planet, affecting worldwide SAT anomalies during warm El Niño and cold La Niña phases^3^. During neutral and La Niña conditions, the subsurface ocean heat accumulates in the tropical western Pacific^4^. While, during El Niño events, the ocean releases heat to the atmosphere, primarily due to increased air-sea heat fluxes driven by elevated sea surface temperatures (SST)^5^. Accordingly, during El Niño phase, enhanced atmospheric heating in the tropics accelerates a rise in global annual mean surface temperature (GMST), contributing to record-breaking warming (e.g., 2015–2016)^6,7^. Conversely, persistent cooling in the eastern Pacific or weak El Niño activity may contribute to a global warming slowdown or hiatus as occurred during 1998–2013^6,8^. 

Global warming exhibits distinct regional patterns^9^. Even during the most recent hiatus period, the record high SATs still occurred in certain regions^7,10^ and it is recognized that a slight elevation in GMST can lead to significant amplification of regional extreme events^11^. Following a rare 3-year 2020–23 La Niña^12,13^, the evolving 2023 El Niño is expected to elevate SATs driven by human-caused climate change^14,15^, and make it more likely that SAT record will be broken worldwide. In fact, during the early stages of the current El Niño development, record-breaking SATs in the boreal summer of 2023 have already led to life-threatening marine and terrestrial heat waves. This study aims to address the prospect of exceptionally high SATs in the upcoming year, in connection with the further progression of the 2023–24 El Niño, and to pinpoint the specific regions where such extremes are expected to occur.

## Results

### A simple model for predicting GMST

On inter-annual timescales, ENSO plays a prominent role in the energy redistribution of the climate system, driven by its strength and extensive global impact^3,5^. The statistical relationship between SST anomalies in the central-eastern tropical Pacific and the unusual heating rate of the tropical atmosphere is nearly linear^6^. As a result, ENSO tightly influences the rate of change in GMST. There are various indices or thresholds to classify the ENSO type (i.e., Central Pacific and Eastern Pacific events) and intensity (i.e., moderate and strong) of ENSO^16–19^. To capture different types of ENSO flavors, an ENSO index (*T*_*NINO*_) is defined by averaging SST anomalies in 160°E–90°W and 5°S–5°N^6^. This index incorporates both the Niño3 and Niño4 region. The high correlation (exceeding 0.9) between the annual-mean *T*_*NINO*_ and the net atmospheric heating anomalies within the tropical Pacific has been confirmed, previously^6^. The intensity levels of El Niño are classified by the annual-mean values of *T*_*NINO*_. ENSO events typically peak in November–January^20^, therefore all annual means in this study are defined from July to June. For instance, the annual-mean SST for 2024 refers to the average of July 2023 through June 2024. According to the standard deviation of yearly *T*_*NINO*_, 14 moderate ($0.9 ^\circ {\text{C}}>{T}_{NINO}\ge 0.6^\circ {\text{C}}$), and 10 strong (${T}_{NINO}\ge 0.9^\circ {\text{C}}$) El Niño years are defined. Those recognized moderate and strong events, such as 1973/74, 1982/83, 1997/98, and 2015/16, can be well distinguished in this way (*see Method* and Fig. S1).

A simplified yet effective, physically-based forecast model of GMST variations, originally developed by Hu and Fedorov^6^, has been adapted and applied in this study. This model is based on a first-order differential equation that effectively describes the rate of change of annual-mean GMST (ΔGMST). It considers various factors, including greenhouse gas emissions, ENSO influences, stratospheric sulfate aerosols produced by volcanoes, and the damping rate of the climate system (see Eq. (1) in the Methods). The coefficients in the model, representing the impacts of the forcing factors on △GMST, are estimated by multiple linear regression. For instance, a regression coefficient of 0.127 year^−1^ for ENSO implies that each 1 ℃ of SST warming in the central-eastern tropical Pacific leads to a 0.127 °C increase in GMST. To ensure the robustness of results and enable ensemble prediction, random re-sampling is also employed to expand the sample size (see Methods and Supplementary Fig. S2).

**Figure 1:**
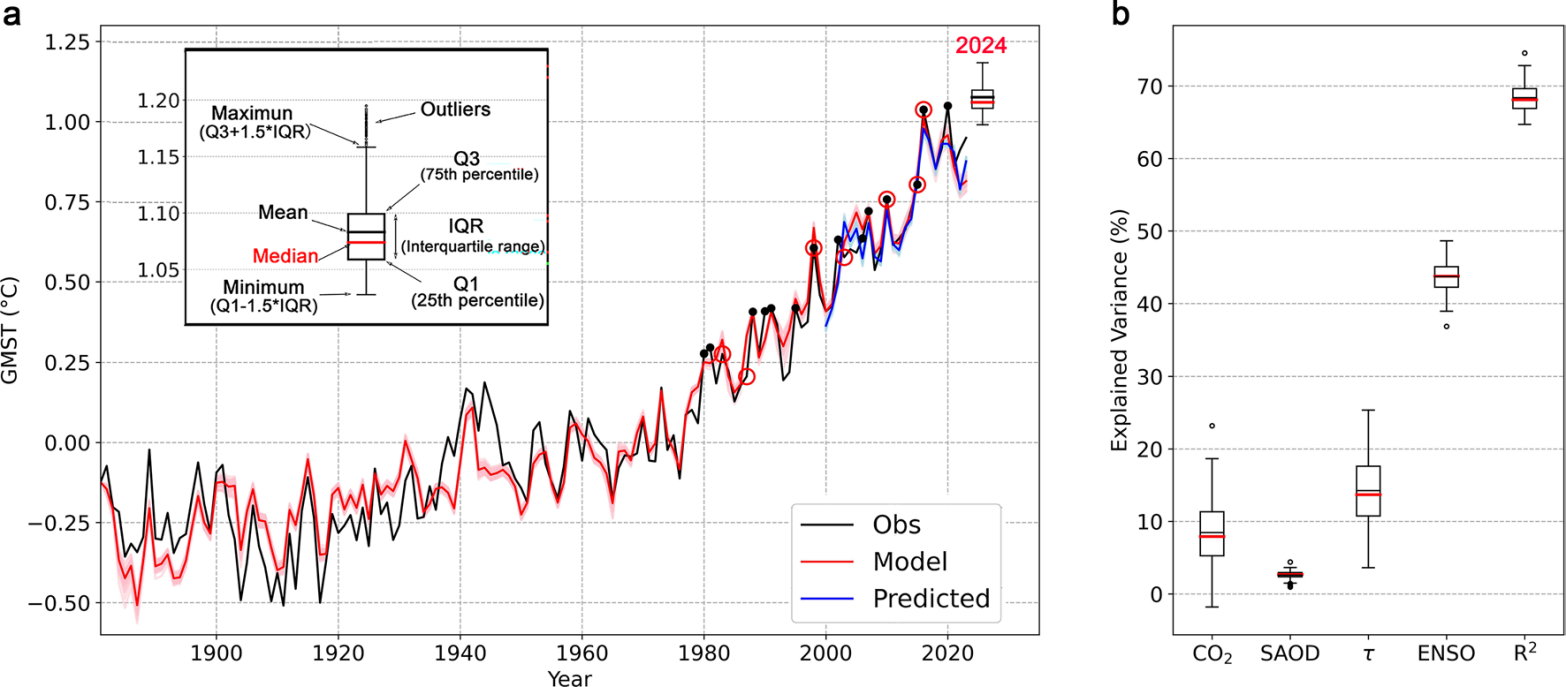
Observed, model-simulated, and predicted GMST variations during the period of 1881–2024. GMST variations are derived from observations, computed from the model, and predicted through forward-rolling experiments (**a**). Black dots (red open circles) in (**a)** indicate the years of record-breaking GMST (moderate and strong El Niño) since 1980. The boxplot illustrates the range of ensemble predictions for 2024. In (**b**), the explained variance of observed GMST changes by all forcing terms (R^2^) and individual forcing terms are displayed.

Next, we evaluated the model’s performance. The model incorporates the term of the damping rate. The effect of the damping rate depends on the previous year’s GMST. When using the observed GMST in the previous year, this straightforward model accurately replicates ΔGMST, with a root-mean-square error (RMSE) of approximately 0.07 ℃ over the instrumental record (Supplementary Fig. S3). While, using an initial GMST value, we can simulate the long-term variations in GMST through model integration. Starting from the second step, the simulated ΔGMST is influenced by the error of the simulated previous year’s GMST. Therefore, the results from the model integration are subject to cumulative errors (Supplementary Fig. S4). Even though, the model can effectively capture the evolution of GMST since 1881 (see Fig. 1).

Furthermore, we conducted forward-rolling predictions for inter-annual forecasting. Taking the hindcast initialized in 1999 as an example, we trained the model using data from 1881 to 1999 and then predicted the GMST in 2000 (as indicated by the blue line in Fig. 1). These results affirm that this simple model is effective in forecasting inter-annual variation of GMST.

The explained variance by individual factors provides insight into GMST changes (see *Methods*). The coefficient of determination (R^2^), a common measure of the goodness of fit to a linear regression, can be interpreted as the proportion of the variance accounted for by the predictands. Approximately 68.4% of the variance in ΔGMST can be accounted for by this simple model (Fig. 1b). We further examined the relative importance of individual forcing factors in contributing to the R^2^ (see *Methods*). Our results reveal that ENSO (43.7%) and the damping rate (14.2%) contribute a combined 57.9% to the total R^2^ value (see Fig. 1b). However, ENSO has limited cumulative influence on the long-term trend of GMST due to its frequent oscillation between cold and warm phases^6^ (see Supplementary Fig. S1). Conversely, CO_2_ significantly affects the long-term trend in GMST (see Supplementary Fig. S1) but has a limited impact on the inter-annual variability of ΔGMST. It is worth noting that the damping rate, as a measure of climate sensitivity, exerts a substantial effect on both the long-term trend^6^ and inter-annual variability of GMST (see Fig. 1b). This rate is characterized by an *e*-folding time scale (see Eq. (1) in Methods) that represents the memory or the persistence of the climate system. In short, year-to-year variations in △GMST are primarily determined by ENSO and the damping rate.

Finally, we can predict GMST in 2024 with this model. Historically, the CO_2_ concentration grows steadily, while stratospheric aerosol optical depth (SAOD) exhibits a small variation in recent decades, whose high values in history are closely associated with episodic volcanic eruptions (see Supplementary Fig. S1). Accordingly, CO_2_ concentration is set to 421.6 ppm, which is estimated by projecting the 2.2 ppm increase during 2022–2023 into 2024; The SAOD in 2024 is set to the same value as in 2023. Considering their limited impact on the inter-annual variability of ΔGMST (Fig. 1b), different assumptions of CO_2_ concentration and SAOD have little effect on the range of the ensemble projections. An El Niño event is expected to persist in 2024, and the observed SST anomalies in the eastern equatorial Pacific in September were greater than 1.5 °C, an intensity comparable to strong historical events^14,15,21^(https://origin.cpc.ncep.noaa.gov/products/precip/CWlink/MJO/enso.shtml#current ). Accordingly, we estimate the probability distribution of El Niño-related atmospheric heating rate from historical observations (*Methods* and Supplementary Fig. S5). Model outputs suggest that under a moderate or strong El Niño scenario, the GMST averaged from July 2023 to June 2024 will likely break the historical record with 90% chance (Fig. 1). The GMST is projected to range from 1.028 ℃ to 1.097 ℃ under a moderate El Niño scenario, while under a strong El Nino scenario, the GMST is projected to range from 1.064 to 1.195 ℃ (Supplementary Fig. S6).

### Outlook for the spatial distribution of record-breaking SAT in 2024

The timescale of inter-annual prediction (i.e. at lead times of 1–2 years), falling between seasonal and decadal prediction, has received relatively limited attention in previous research^22,23^. Inter-annual prediction skills using initialized numerical coupled climate models with large ensembles appear to be notably lower over continental land regions, such as Asia, and this skill diminishes further when trends linked to global warming are factored out^23^. This suggests that state-of-the-art climate models may have constraints in forecasting year-to-year changes and predicting the spatial patterns that lead to record-breaking events. To predict the spatial distribution of surface air temperature (SAT) on a global scale, we have extended our GMST forecast model to encompass each 2° × 2° grid point across the globe. The rate of SAT change (ΔSAT) for each grid is determined through a first-order differential equation, with coefficients estimated using multiple linear regression (see Eq. (1) in the Methods section). This model operates as a patchwork, independently simulating SAT for each grid point and then amalgamating them to create a spatial distribution.

**Figure 2:**
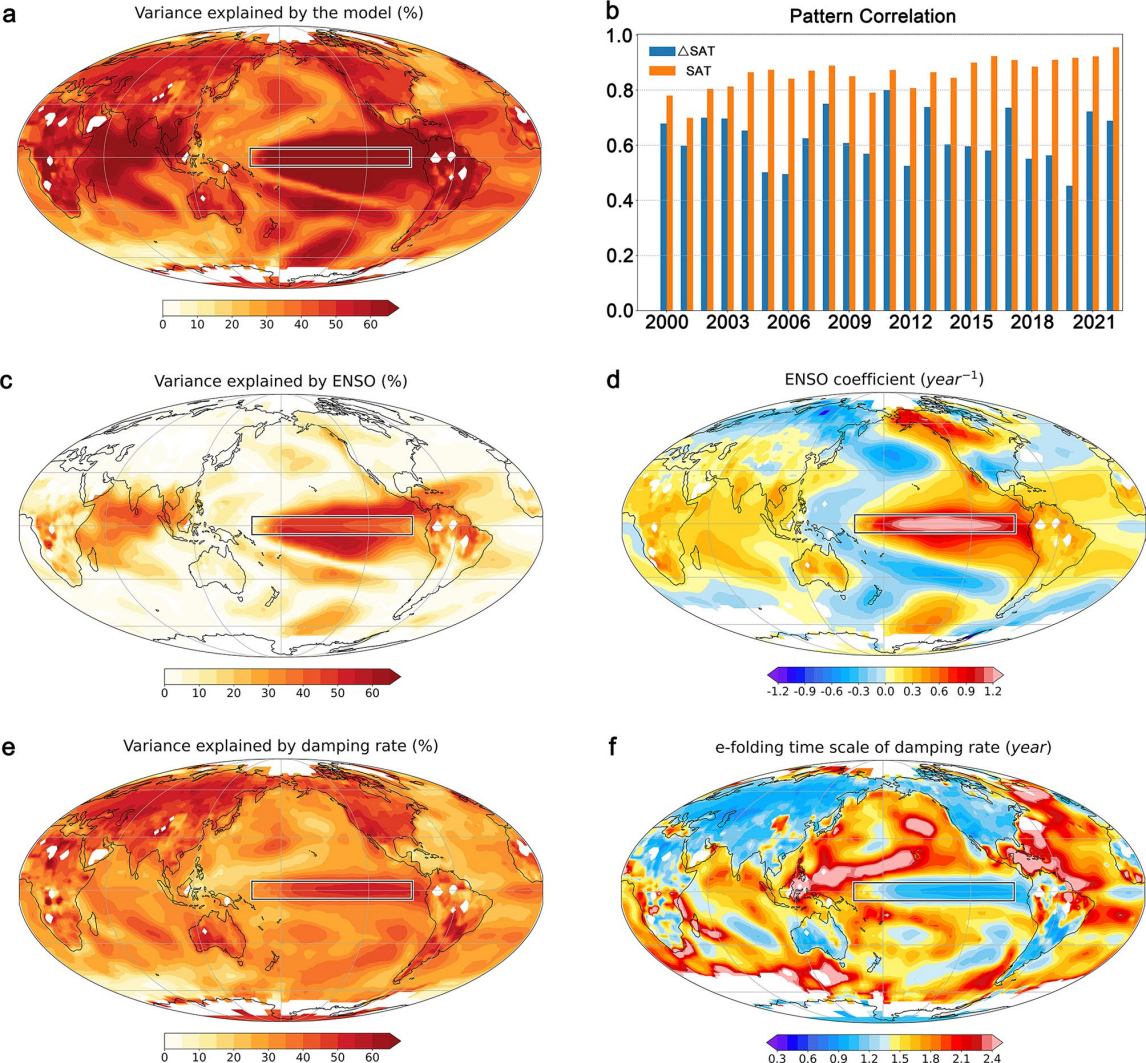
Model forecast performance and dominant forcing factors. (**a**). The explained variances of observed ΔSAT by the model (R^2^). (**b**). The ΔSAT and SAT pattern correlations between forward-rolling predictions and observations since 2000. **c.** and **e**. the explained variances contributed by ENSO and the damping rate, respectively, similar to the map in (**a**). Regression coefficients for ENSO and damping rate are shown in (**d**) and (**f**). The black box denotes the region used for the ENSO index.

The model effectively reproduces ΔSAT at most grid points worldwide, except for polar and sub-polar regions (see Fig. 2). Additionally, we perform a forward-rolling prediction experiment, where ΔSAT, as an inter-annual increment, served as the direct predictive target (see *Methods*). The final predictions for the SAT distribution were derived from the current state plus the increments. Remarkably, even with a linear model comprising only four factors, the pattern correlation between the predicted ΔSAT and observed values stands at approximately 0.63, while the pattern correlation for the SAT distribution reaches around 0.86 (see Fig. 2b). A skill map is illustrated in the supplement (see Supplement Fig. S7).

Comparing the relative importance of the different factors (see *Methods*), we note that ENSO and the damping rate are the primary drivers of SAT variations across the globe (see Fig. 2), consistent with the GMST model. ENSO exerts a significant influence on SAT variability in the tropics (see Fig. 2c), while the damping rate dominates in the subtropics, particularly over continental regions (Fig. 2d). The distribution of regression coefficients for ENSO (see Fig. 2d) and the damping rate (see Fig. 2f) illustrate their regional impacts on SAT. Unlike the impacts of the radiative forcing factors, which exhibit minimal spatial variations (see Supplement Fig. S8), ENSO and damping rates show significant spatial structures that affect SAT variations.

These ENSO-related teleconnections have been extensively studied and are consistent with previous publications^3,5^. In contrast, the role of the damping rate, particularly its regional impacts, is more intricate and demands further investigation. The damping rate, described by an *e*-folding time scale (as per Eq. (1) in Methods), represents the memory or the persistence of the climate anomalies from one year to the next. Climate persistence spans a range of timescales, from months to decades^24–26^. The multi-year to decadal memory of the climate system, resulting from complex interactions across various climate subsystems, has been a focal point in the study of global climate change^9,27^.

In the context of the ENSO timescale, our results indicate that inter-annual climate memory is associated with local intrinsic characteristics, such as heat capacity. Notably, a prominent feature of the distribution of inter-annual climate persistence is the distinction between land and sea (see Fig. 2f). The ocean’s higher persistence primarily results from its thermal inertia and slower variation modes. Consequently, regions with a maritime influence exhibit considerably stronger climate memory than inland areas. Oceanic areas with significant persistence are primarily concentrated in the tropical Pacific and Atlantic warm pools, characterized by deep oceanic thermoclines that store substantial amounts of warm water. On the other hand, the far northwestern Atlantic SST influenced by AMO shows strong year-to-year persistence but lacks a clear warming trend^28^. Over the continents, temperature changes in East Asia exhibit stronger persistence compared to North America (see Fig. 2f). Owing to the stronger persistence, the warming rate of SAT over China is significantly (1/3–1/2) higher than that over the United States^9^. In summary, the year-to-year persistence of climatic conditions means that present anomalies strongly influence the following year’s climate state, and the spatial structure of persisted anomalies plays a crucial role in distinct regional SAT variations.

**Figure 3:**
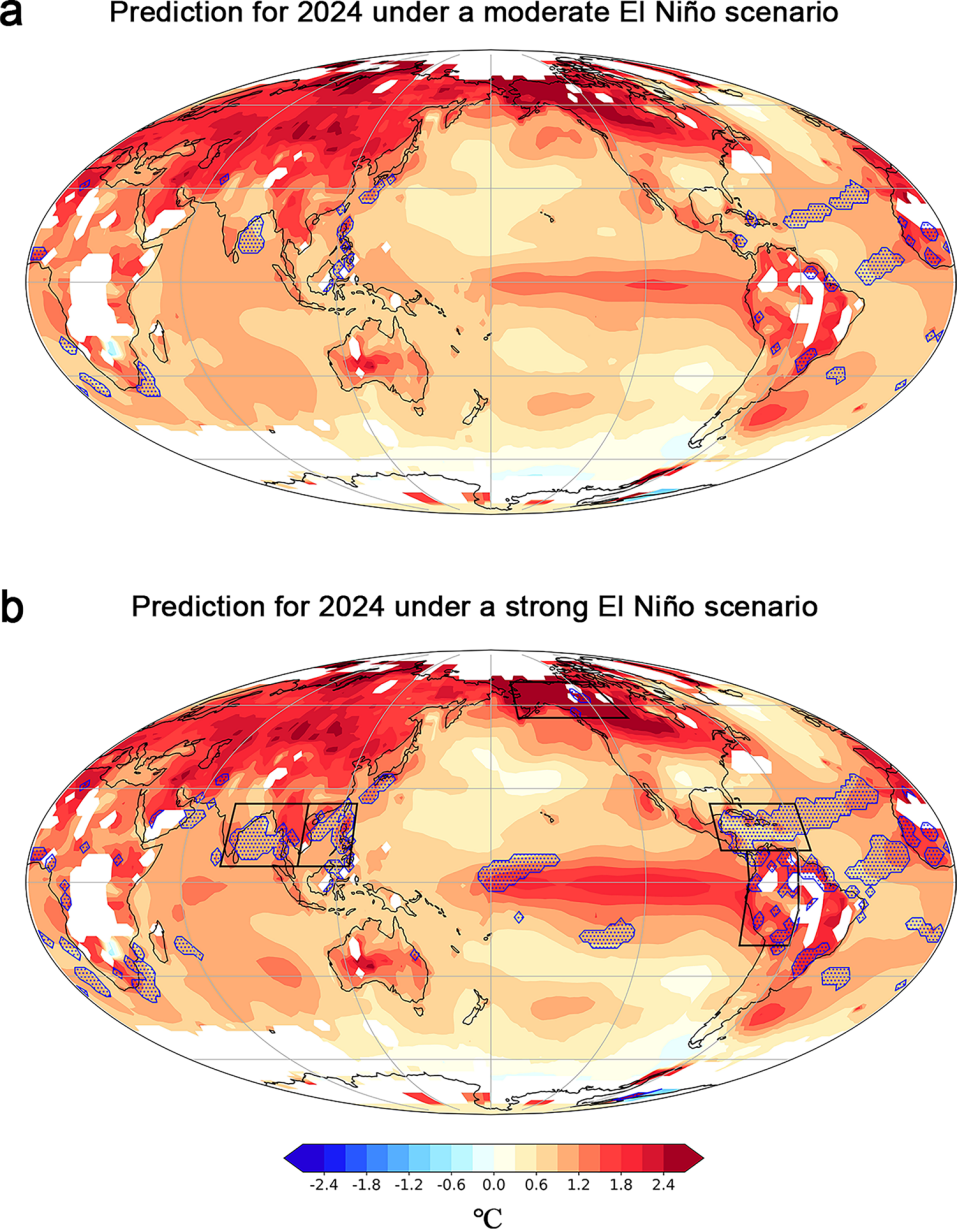
Predicted global SAT variation for 2024 (color shaded) under (**a**) a moderate El Niño scenario and (**b**) a strong El Niño scenario In both (**a**) and (**b**), the regions with record-breaking heating are marked by blue dots. Black boxes in (**b**) note the regions: the Bay of Bengal (5°N–25°N, 75°E–105°E), the South China Sea (5°N–25°N, 105°E–125°E), the Caribbean Sea (10°N–25°N, 55°W–90°W), Alaska (55°N–70°N, 105°W–165°W), and the Amazon (20°S–10°N, 60°W–80°W).

Finally, the model generates global SAT distribution for 2024 under varied El Niño strength scenarios (Fig. 3). These results reveal substantial SAT anomalies that are primarily centered in extratropical continental regions. Record-breaking SAT, on the other hand, are primarily anticipated in coastal and adjacent seas, encompassing regions in Southeast Asia, South Africa, Alaska, northern South America, and the tropical Atlantic.

### Implications of the results

**Figure 4:**
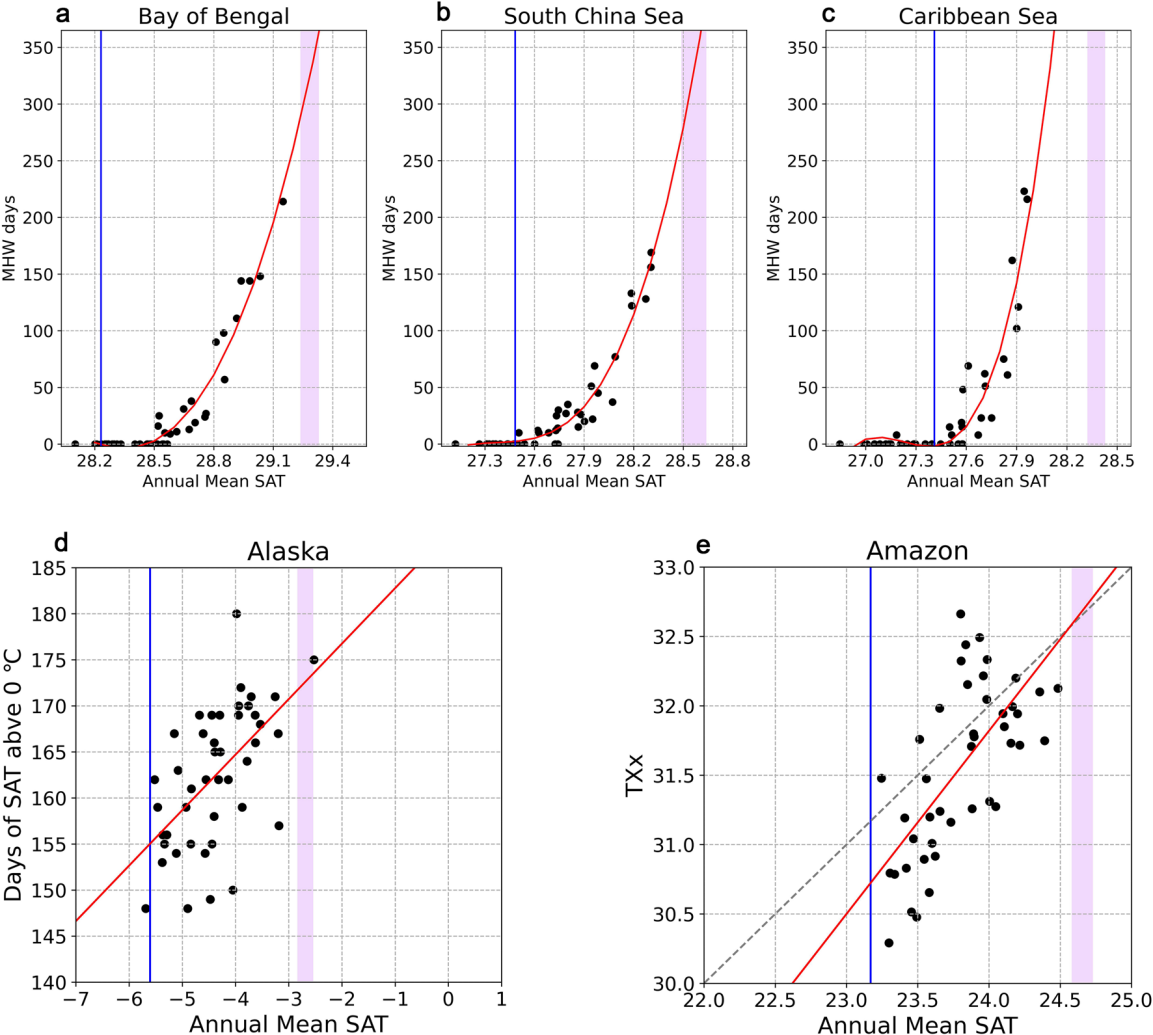
Changes in regional extremes as function of annual SAT. (**a**–**c**) The annual marine heatwave (MHW) days (*see Methods*) for the Bay of Bengal, the South China Sea, and the Caribbean Sea. (**d**) The number of annual days with daily mean SAT greater than 0 ℃ in Alaska. (**e**) The annual maxima of daily maximum SAT (TXx) for the Amazon. The regions are marked by black boxes in Fig. 3b. The black dots are the raw values; the red lines are the regression lines. The blue lines are the reference lines, corresponding to 1951–1980 means. The dashed diagonal lines in (**d**) and (**e**) are reference lines with slopes equal to 1. Columns in light pink represent the ranges of the predicted regional SATs between the moderate and strong El Niño scenarios in Fig. 3.

Firstly, our results point to the likelihood of record-breaking GMST between July 2023 and June 2024, primarily driven by a developing moderate to strong El Niño event. Additionally, we offer insight into the spatial distribution of surface air temperature (SAT) and the regions where record-breaking temperatures may occur. Elevated temperatures can lead to a significant increase in the likelihood of extreme events and risks from a range of natural hazards^29,30^. For instance, the possibility of record-breaking SST in the Bay of Bengal, the South China Sea, and the Caribbean Sea would potentially lead to year-round marine heatwaves (see Fig. 4a–c), resulting in negative ecological, economic, and social consequences^31–33^. The warming in Alaska (see Fig. 4d) would result in a series of negative responses or feedbacks, including glacier and permafrost melting, coastal erosion, and other negative climate impacts^34^. The record-breaking SAT in the Amazon may worsen extreme weather (see Fig. 4e), increasing wildfire risk. In fact, severe wildfires and drought have already hit the Amazon this past September and October 2023 (https://earthobservatory.nasa.gov/images/151965/drought-fuels-wildfires-in-the-amazon).

Secondly, we found that, compared to greenhouse gas forcing, ENSO can lead to appreciable year-to-year fluctuations in GMST. Strong El Niño events can cause GMST to rise rapidly, potentially exceeding the preferred ambitious 1.5 ℃ target of the Paris Agreement^35^ for a short period; multi-year La Niñas can slow down global warming^36^. Thus, the impacts of ENSO variability on GMST are a more urgent concern for inter-annual variations in GMST^37^.

While climate persistence remains a complex and not easily modeled factor^9^, it plays a pivotal role in the inter-annual variability in SAT. The greater persistence of the oceans would accelerate the risk of coastal zones, including more intense heat waves and tropical cyclones^38–41^. Combined with anthropogenic global sea level rise^41^, densely populated coastal areas are facing an enormous and urgent climate crisis that challenges our current capacity for adaptation, mitigation, and risk management.

## Methods

### Surface air temperature data

For global temperatures spanning the period of 1880–2023, we use monthly Goddard Institute for Space Studies (GISS) Surface Temperature Analysis with a 1200 km smoothing (2° × 2° grid) to calculate the GMST [GISTEMP Team, 2023: GISS Surface Temperature Analysis (GISTEMP), version 4. NASA Goddard Institute for Space Studies. Dataset accessed 2023-07-30 at https://data.giss.nasa.gov/gistemp/]^42^. To evaluate ENSO variations, we use Extended Reconstructed Sea Surface Temperature v5 SST product (https://psl.noaa.gov/data/gridded/data.noaa.ersst.v5.html)^43^. The anomalies of the variables are deviations from the corresponding 1951–1980 means. To calculate marine heatwaves, we use daily SSTs with 1/4 resolution for 1982–2023 from the National Oceanic and Atmospheric Administration optimum interpolation SST (OISST v2) dataset^44^ (https://psl.noaa.gov/data/gridded/data.noaa.oisst.v2.html). To calculate the annual maxima of daily maximum SAT (TXx) over the continents, we use the Climate Prediction Center (CPC) 0.5° × 0.5° Global Daily Gridded Temperature Dataset for 1979–2023 (https://psl.noaa.gov/data/gridded/data.cpc.globaltemp.html).

### Radiation forcing data

For CO_2_ concentrations, we use Mauna Loa in situ measurements downloaded from the NOAA Earth System Research Laboratory (ESRL) website (www.esrl.noaa.gov/gmd/ccgg/trends/) available after 1959, combined with ice core reconstructions from Law Dome DE08 and DE08-2 (http://cdiac.ornl.gov/ftp/trends/co2/lawdome.smoothed.yr20) before that^6^.

To account for volcanic eruptions, we employ a stratospheric aerosol optical depth (SAOD) data set from NASA GISS (https://data.giss.nasa.gov/modelforce/strataer/tau.line_2012.12.txt) during 1850–2012^45^. To extend the period of the data set to present, we have also used the datasets from a combined SAOD time series during 1850–2019 (10.5281/zenodo.4300780)^46^ and the afterward observation from MODIS Aqua (https://giovanni.gsfc.nasa.gov/giovanni). The relationship between SAOD and the latter two was established by linear regression during the overlapping periods. Based on these relationships, we construct a time series of SAOD during the period of 1850-present (Supplement Fig. S1).

### ENSO index

Following the study of Hu and Fedorov^6^, an ENSO index, *T*_*NINO*_, is defined by averaging SST anomalies within a large equatorial Pacific domain between 5°S–5°N and 160°E–90°W. This index incorporates both the Niño3 and Niño4 regions (the black box in Fig. 2a). All annual means in this study are defined for July–June, rather than the calendar January– December. The impacts of the radiative forcing on ENSO are first linearly removed from the ${T}_{NINO}$ index before using in the regression equation (Supplement Fig. S1c). Further, 21 weak ($1.0\sigma >{T}_{NINO}\ge 0.5\sigma$), 14 moderate ($1.5\sigma >{T}_{NINO}\ge 1.0\sigma$), and 10 strong (${T}_{NINO}\ge 1.5\sigma$) El Niño years are defined. The standard deviation ($\sigma$) of ${T}_{NINO}$ in Fig. S1c is 0.6.

### Modeling of GMST and SAT distribution

The model incorporates the main factors affecting GMST and is based on a first-order differential equation describing the rate of change of annual mean GMST:

1 $$\frac{d{T}_{g}}{dt}=-\frac{{T}_{g}}{\tau }+a\cdot {\text{ln}}({CO}_{2}/{CO}_{2,ref})+b\cdot {T}_{NINO}+c\cdot SAOD+d$$

The particular terms on the right-hand side of the equation describe (i) linear damping with an *e*-folding time scale τ, (ii) longwave radiative forcing due to greenhouse gases (mainly carbon dioxide, CO_2_, ref = 320 ppm.), (iii) atmospheric heating anomalies associated with ENSO (positive into the atmosphere and assumed proportional to *T*_*NINO*_), (iv) shortwave scattering by stratospheric sulfate aerosols induced by volcanic eruptions, and (v) a residual term, respectively. 1 year is used as the time step. ${T}_{g}$ stands for annual mean GMST or each grid’s SAT.

### Random re-sampling for regression

The regression coefficients in Eq. (1) may depend on the period chosen. To estimate the ranges of the coefficients, we employed random sampling to expand the sample size, ensuring the robustness of results and enabling ensemble predictions. We performed 50 randomized re-sampling experiments, each time dividing the dataset for the entire period into random training and testing subsets. The proportion of the dataset to include in the train split is set to 80%. As a result, we constructed 50 models with different regression coefficients. When combined with the historical probability distribution of *T*_*NINO*_ (24 above moderate events: 14 moderate and 10 strong events), this led to an ensemble prediction consisting of 1200 members, as shown in the boxplot in Fig. 1). The distributions of the regression coefficients and their changes associated with different test-train split proportions are illustrated in Supplementary Fig. S2.

### Relative importance analysis of the forcing factors

The coefficient of determination (${R}^{2}$), a common measure for the goodness of the fit for linear regression, can be interpreted as the proportion of the variation of the predictand. A better predictor has *R*^2^ closer to 1.

Suppose there is a multiple linear regression equation as follows:

2 $$y={\sum }_{j=1}^{J}{a}_{j}{x}_{j}+e=\widehat{y}+e$$

${a}_{j}$ are the regression coefficients and $e$ stands for the residual. ${\text{y}}$ is the true value of the predictand and $\widehat{y}$ is the predicted or explained value by the explanatory variable ${x}_{j}$. ${R}^{2}$ can be expressed as:

3 $${R}^{2}=\frac{RSS}{TSS}=\frac{Var(\widehat{y})}{Var(y)}=1-\frac{Var(e)}{Var(y)}$$

$RSS$ is the regression sum of squares, and TSS is the total sum of squares.

When dealing with multiple predictors, a fundamental question arises: which of these predictors is most crucial or effective in predicting the outcome variable? It is evident that when no correlation exists among the explanatory variables, a straightforward method to gauge the significance of each predictor is by calculating its covariance with the explained variable and then dividing this value by the variance of the explained variable. This approach provides a clear measure of each variable’s contribution.

4 $${R}^{2}=\frac{{\sum }_{j=1}^{J}{a}_{j}Cov({x}_{j},y)}{Var(y)}$$

## Definition of marine heatwaves

We identified MHWs from daily SST time series following ref.^47^ as a discrete prolonged anomalously warm water event. ‘Discrete’ was defined quantitatively as an identifiable event with recognizable start and end dates, ‘prolonged’ meant a duration of at least 5 days, and ‘anomalously warm’ was defined by reference to a baseline, seasonally varying threshold. Heatwave events were found by identifying periods when daily temperatures were above the seasonally varying 90th percentile (threshold) for at least five consecutive days. The 90th percentile was calculated for each calendar day using daily SSTs within an 11-day window centered on the date across all years within the climatology period and smoothed by applying a 31-days moving average. A seasonally varying threshold allows the identification of anomalously warm events at any time of the year. The days within the start and end dates of an event were defined as MHW days. Accordingly, the total number of MHW days in each year was calculated. The MHW definition as used in this manuscript is available as software modules in Python (http://github.com/ecjoliver/marineHeatWaves) and R (https://github.com/ajsmit/RmarineHeatWaves).

### Supplementary Information

Supplementary Figures.

## Data Availability

The data supporting the findings of this study are available online (https://data.giss.nasa.gov/gistemp/; https://psl.noaa.gov/data/gridded/data.noaa.ersst.v5.html; https://psl.noaa.gov/data/gridded/data.noaa.oisst.v2.html; https://psl.noaa.gov/data/gridded/data.cpc.globaltemp.html; www.esrl.noaa.gov/gmd/ccgg/trends/; http://cdiac.ornl.gov/ftp/trends/co2/lawdome.smoothed.yr20; https://data.giss.nasa.gov/modelforce/strataer/tau.line_2012.12.txt; 10.5281/zenodo.4300780; https://giovanni.gsfc.nasa.gov/giovanni).

## Code availability

The codes used in this study can be download here: http://github.com/ecjoliver/marineHeatWaves.
