# Supplementary material for: Enhanced risk of record-breaking regional temperatures during the 2023–24 El Niño
Source: Sci Rep. 2024 Feb 29;14:2521. doi: 10.1038/s41598-024-52846-2 (PMC10904789; doi:10.1038/s41598-024-52846-2)
Supplement: Supplementary file 1 — Supplementary Figures. [file 41598_2024_52846_MOESM1_ESM.pdf]

Supplementary Material

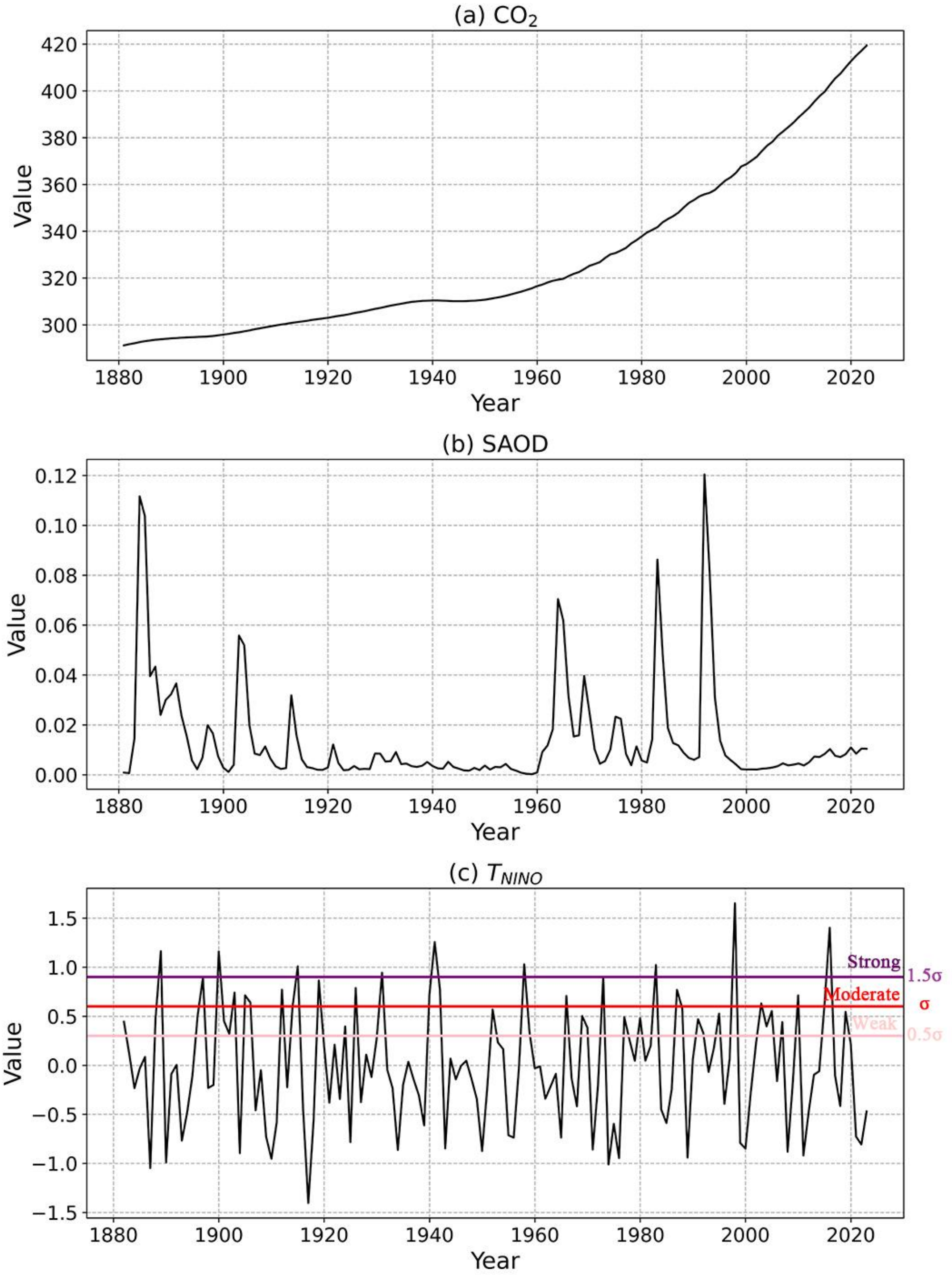

Fig. S1. Time series of individual forcings. (a) Atmospheric CO_2 concentrations (ppm). (b) Stratospheric aerosol optical depth (SAOD). (c) Annual-mean variations in T_{NINO} (°C). The impact of CO_2 and SAOD on T_{NINO} is removed in (c).

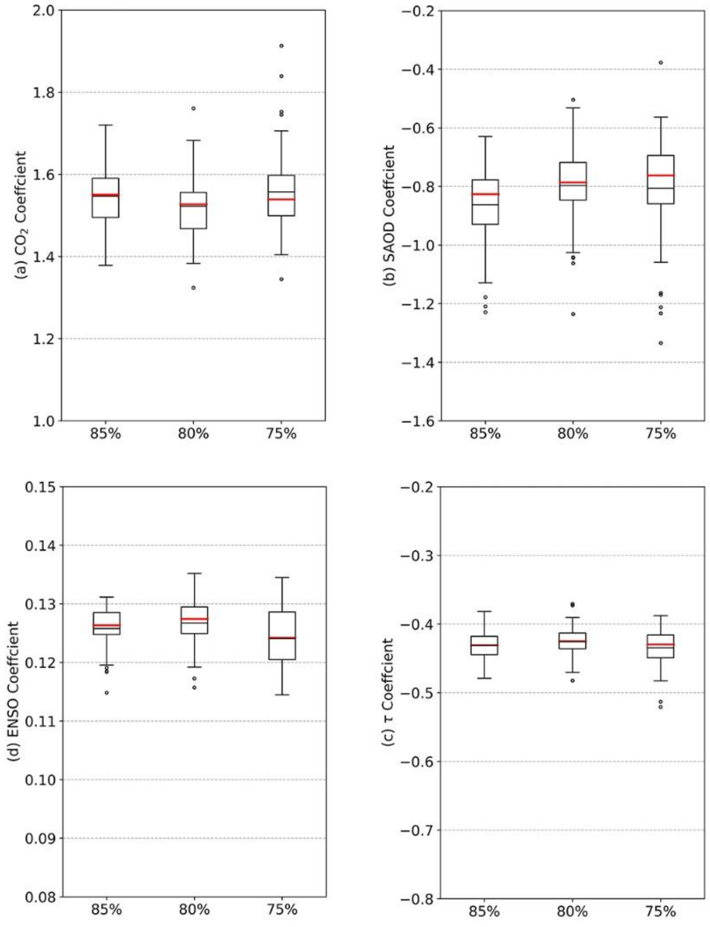

Fig. S2. The boxplots illustrate the ranges of coefficients for (a) CO_2, (b) SAOD, (c) ENSO, and (d) the damping rate with different test-train split proportions. The proportions of the dataset to include in the train split are set to 75%, 80% and 85%.

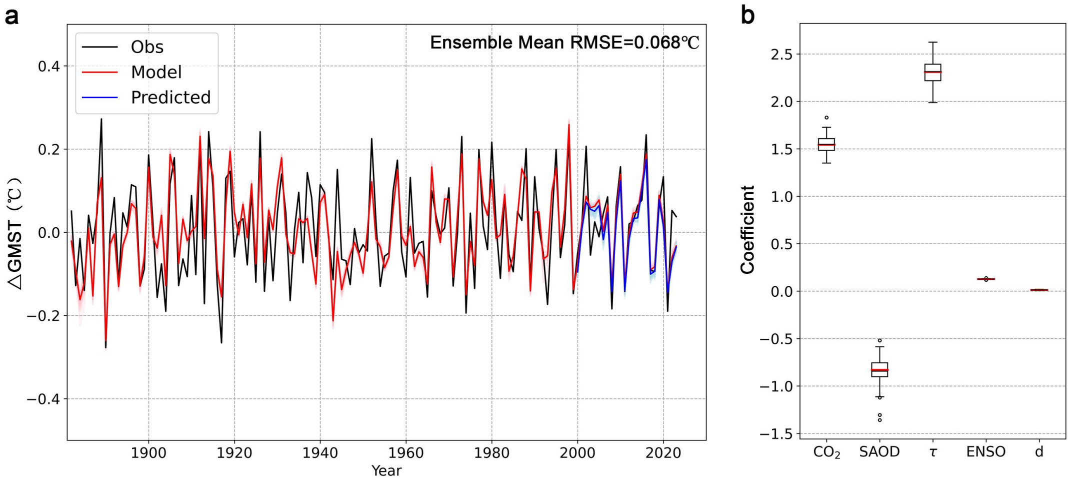

Fig. S3. Observed and model simulated ΔGMST variations. ΔGMST variations are

estimated from observation, computed from the model, and predicted by forward-rolling prediction experiments (a). The regression coefficients for different forcing terms and intercepts are plotted in (b).

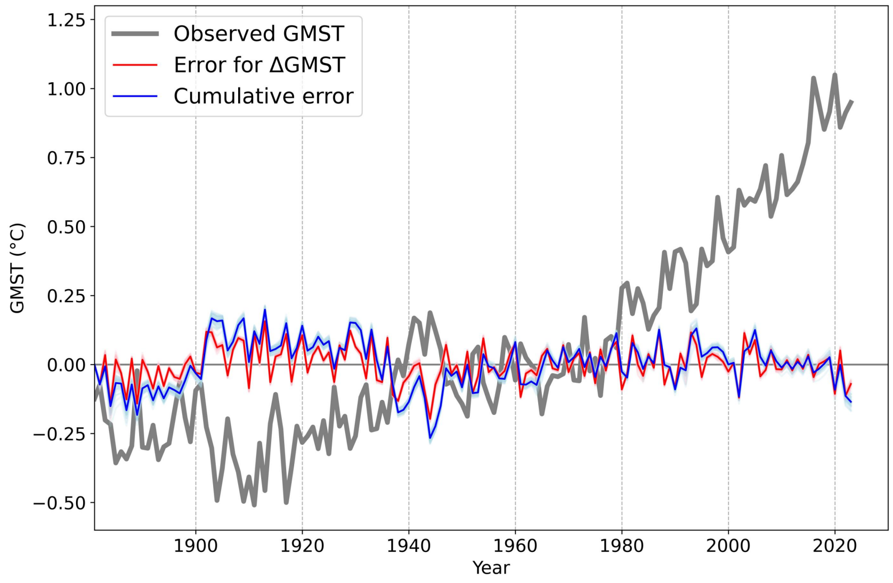

Fig. S4. The differences between the observation and the model simulations. The thick gray line indicates the observed GMST. The red line represents the errors in ΔGMST variations computed from the model in Fig. S3. The blue line represents the errors in GMST variations computed from the model integration since 1881 in Fig. 1.

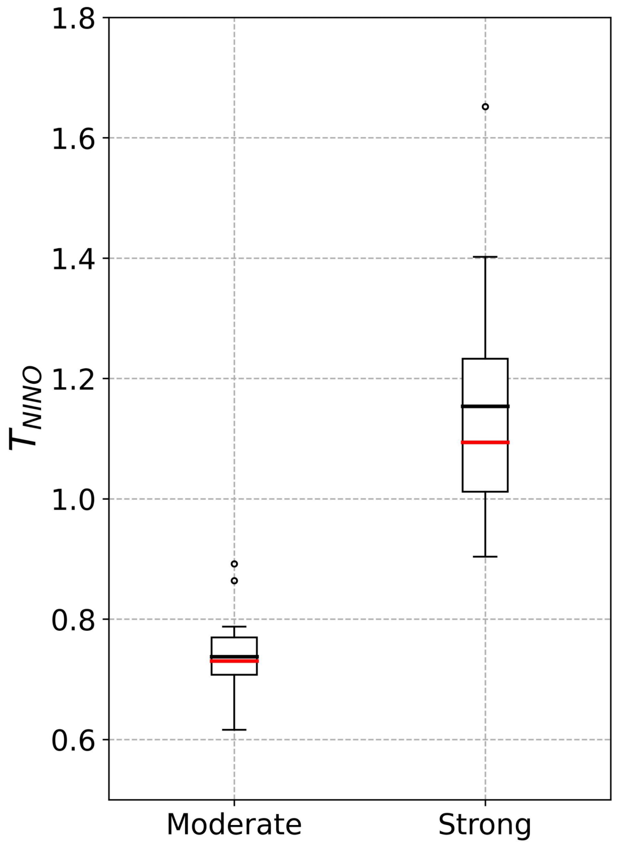

Fig. S5. The distributions of T_{NINO} values for the historical moderate and strong El Niño events.

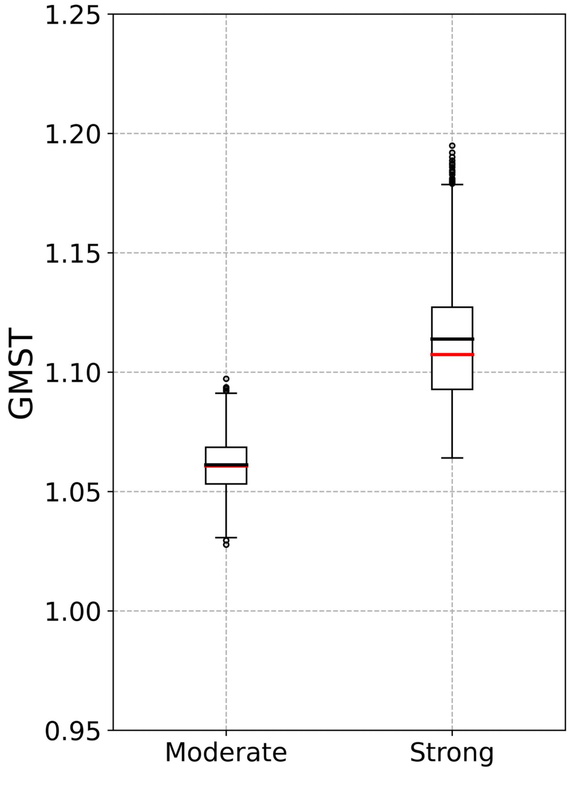

Fig. S6. The distributions of predicted GSMT values for 2024 under moderate and

strong El Niño scenarios.

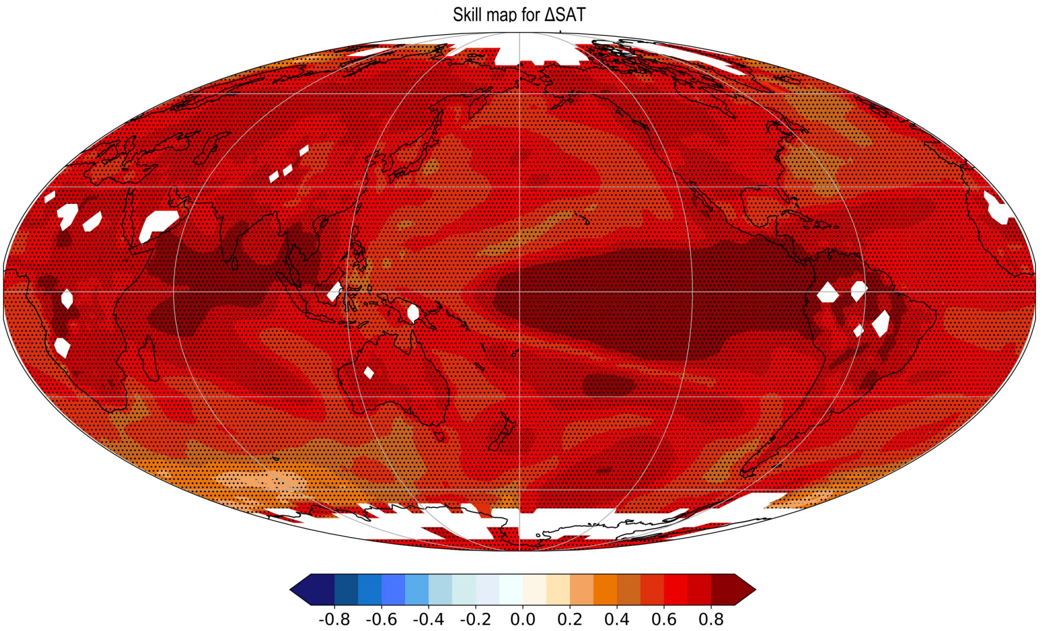

Fig. S7. Skill map for predicting annual-men Δ SAT, stippling shows where correlations are significant at the 95% confidence level.

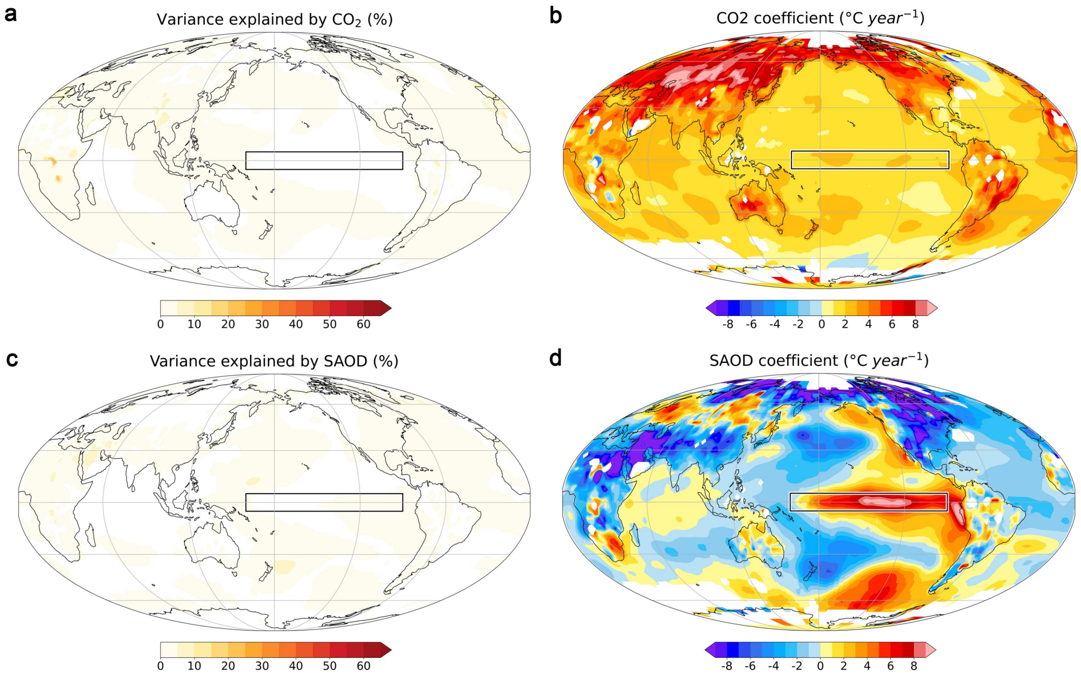

Fig. S8. Similar to Fig. 2, but for the explained variances of the observed ΔSAT by (a) CO_2 and (c) SAOD, respectively. The regression coefficients for CO_2 and SAOD are shown in (b) and (d).
